# Age at Menarche and Risk of Multiple Sclerosis: Current Progress From Epidemiological Investigations

**DOI:** 10.3389/fimmu.2018.02600

**Published:** 2018-11-13

**Authors:** Xia Jiang, Tomas Olsson, Lars Alfredsson

**Affiliations:** ^1^Program in Genetic Epidemiology and Statistical Genetics, Harvard T. H. Chan School of Public Health, Boston, MA, United States; ^2^Neuroimmunology Unit, Center for Molecular Medicine, Department of Clinical Neuroscience, Karolinska Institute, Stockholm, Sweden; ^3^Cardiovascular group, Institute of Environmental Medicine, Karolinska Institute, Stockholm, Sweden

**Keywords:** puberty, age at menarche, hormone, multiple sclerosis, population-based, epidemiology

## Abstract

Multiple sclerosis (MS) is a chronic autoimmune inflammatory disorder of the brain and spinal cord in which focal lymphocytic infiltration leads to the damage of myelin and axons. As a multi-factorial complex trait, both genetic background and environmental factors are involved in MS etiology. The disease is more prevalent among women, and an overall female-to-male sex ratio of around 3 is usually reported. The fact that the female preponderance is only apparent among patients with disease onset after age 12 points toward a role of puberty in MS. A key marker of female pubertal development is menarche, however, evidence from previous epidemiological investigations has been sparse and conflicting: although some studies have linked earlier age at menarche (AAM) to an increased risk of MS, others have found no association or an inverse association. Understanding the effect of AAM in MS could increase our knowledge to the disease etiology, as well as deliver meaningful implication to patients' care by aiding clinical diagnosis. Therefore, we reviewed all the currently available epidemiological studies conducted for AAM and *risk of MS* in *adult human populations*. We found evidence supporting a possible favorable role of late AAM on MS risk, but this should be further confirmed by well-designed large-scale epidemiological studies and meta-analysis. Future work may be focused on Mendelian randomization analysis incorporating genetic markers to provide additional evidence of a putative causal relationship between AAM and MS. More work should be conducted for non-European populations to increase generalizability, and among the males to complementary with results from females. Future work may also be conducted focusing on hormonal reproductive factors other than menarche, and their effects in MS prognosis, severity, and drug response.

## Introduction

Multiple sclerosis (MS) is primarily an autoimmune inflammatory disorder of the central nervous system (CNS), characterized by the loss of myelin and damage of axons, leading to a variety of neurological deficits (1). The early course of MS is characterized by episodes of neurological dysfunction that usually recover. However, as the disease progresses, pathological changes become dominated by widespread microglial activation associated with extensive neurodegeneration and accumulated disability. Based on data from WHO, it is estimated that more than two million people (~2.3–2.5 million) worldwide are living with MS and the disease is one of the most common causes of neurological disability (loss of motor and sensory function) among young adults (2). The incidence of MS varies in different regions of the globe, but usually grows with latitudes, making northern Europe a high-risk zone (3, 4). MS strikes women two to three times more often than men, and the female-to-male sex ratio keeps rising (5). While women carry higher disease risk, they usually present less rapid progression to disability (disease severity).

MS is a complex disease with both genetic and environmental factors involved in its etiology. To date, over 200 associated loci have been identified through genome-wide association studies (GWAS) (6), and a low but steadily increasing number of environmental risk factors such as cigarette smoking, EB-virus infection, vitamin D insufficiency (and more) have been consistently observed to be associated with MS risk (7, 8). It has also been long documented that hormonal related factors are crucial in the disease susceptibility and development. Evidence from animal models (experimental allergic encephalomyelitis, EAE) has shown that estrogen, progesterone (at pregnancy range) and testosterone provide anti-inflammatory and neuroprotective effects on both the induction and effectors phases (9). The fact that female preponderance is only apparent among patients with age of disease onset after 12 (female-to-male sex ratio for pediatric MS before 12: 1.2:1) (10) points toward a role of puberty in MS predisposition. A key marker of female pubertal development is menarche, indicating an initiation of a reproductive life. Despite several lines of evidence from epidemiological investigations linking earlier age at menarche (AAM) to an increased risk of MS, other studies have found no association or an inverse association. Most of the previous observational studies have been small, or conducted in selected clinical samples, yielding highly variable estimates. MS is most commonly diagnosed among women who are 20–50 years old and of child-bearing age. It is thus not surprising that patients are usually interested in the topics of pubertal changes, motherhood, and disease.

In this review, we will summarize the results from epidemiological studies conducted so far investigating the relationship between age at menarche and risk of MS in human adults (we collected all the relevant articles through an electronic search from PubMed, with key words “puberty,” “pubertal,” “menarche,” “hormone,” and “multiple sclerosis.” Only published data were included). We will comment on the advantages and limitations of these studies. We will conclude by presenting challenges, current research gaps and potential future directions for this field. We anticipate that the increased knowledge regarding the association between age at menarche and MS risk as presented by our review will provide insights into the mechanistic developmental processes of MS, as well as facilitate patient care and women's health by aiding clinical consultations.

## Age at menarche and risk of MS–results from early retrospective studies

Female menstruation and the risk of MS was first examined by Antonovsky et al. (11) using a population-based case-control study in Israel consisting of 241 patients and 964 controls (of which, 131 female cases and 523 female controls). They found a significantly shorter self-reported menstruated period (the length of menstruation, usually lasts about 3–5 days) among the cases than controls (*P* = 0.01 for the Chisq-test), but no differences were observed for age at menarche or mean length of menstrual cycle (the length of menstrual cycle, usually 28 days but can be varied from 21 to 35 days). The study had a population-based design where the patients were drawn from a nationwide survey of MS (participation rate: 92%) and controls were randomly selected from the population registry of census, matched on age and sex (case-control ratio 1:4). Study participants answered a questionnaire containing 146 questions covering 11 major areas. The participants were from an admixed population consisting of five regions of birth (Eastern Europe, Central-western Europe, Southern Europe, Asia-Africa, and Israel). The heterogeneous genetic background was not taken into account in the study, which might influence the effect of menstruation (11).

The null finding concerning age at menarche was contrasted by two subsequent small case-control studies carried out in 1989 in European ancestry populations. One was conducted by Berr et al. in France which included 63 prevalent cases and 63 controls (46 female case-control pairs). A statistically significant older mean age at menarche was observed among patients than among controls (13.5 vs. 12.7, *P* < 0.002 for the *t*-test) (12). The other was conducted by Operskalski et al. in USA which composed of 145 cases and 145 controls (108 female case-control pairs), where an inverse association was observed; the cases were significantly younger at menarche than controls (12.3 vs. 12.7, *P* = 0.01 for *t*-test) (13). The conflicting results from these two studies are perhaps not surprising, as both studies were small.

Nevertheless, findings from two recent studies supported again the previous non-significant association between age at menarche and MS as identified by the Antonovsky et al. study. Kurtzke et al. examined 23 MS patients and 127 controls in UK, and reported age at menarche did not differ significantly between cases and controls (13.6 vs. 14.0), although the cases presented a slightly younger AAM. However, this result was based on extremely underpowered data with only 9 female cases and 68 female controls from a selected veteran population (14). Similarly, Gustavsen et al. compared 391 cases and 535 controls in Norway (all female), and found age at menarche did not differ significantly between the two groups (13.07 vs. 12.97), although the cases showed a slightly older AAM (15).

To summarize, these earlier epidemiological studies were mostly underpowered with limited sample size ranging from 9 to 391 cases, yielding highly variable estimates. All studies used self-reported prevalent cases and self-administrated questionnaires for the collection of exposures, introducing a potential recall bias. Moreover, these studies lacked proper design, as most of them did not explicitly studied AAM, but rather collected this exposure complementary to other main exposures of interest. Thus, the numbers of questions in terms of hormonal related factors and AAM are limited and unspecific; and very little information is provided on how the questions were formulated. Finally, none of the aforementioned studies performed a formal statistical analysis with adequate control for confounding factors, but rather performed a simple comparison between the cases and controls using a *t*-test or a chisq-test, which may yield biased results. More details are presented in Table 1. Despite all the limitations and inconclusive findings, these earlier studies provide some preliminary support for a role of AAM in the risk of MS.

**Table 1:** The epidemiological investigations on age at menarche and risk of MS, results from early retrospective studies.

**Author**	**Year**	**Population**	**Study**	**Design**	**Sample size**		**Mean age at menarche**		***P*-value**	**Limitations**
					**Cases/controls**	**Female subjects**	**Cases**	**Controls**		
Antonovsky et al.	1965	Mixed	Case-control study	Population-based	241/964	131/523	Data not reported	Data not reported	Not significant; *P*-value not reported	1. Data not shown; 2. simple Chisq test; 3. admixed populations; 4. Self-reported definite or probable case; 5. prevalent case.
Berr et al.	1989	Caucasian	Case-control study	Population-based	63/63	46/46	13.5	12.7	*P* < 0.002	1. Small sample size (<100 cases); 2. simple two sample *t*-test; 3. prevalent case.
Operskalski et al.	1989	Caucasian	Case-control study	Population-based	145/145	108/108	12.3	12.7	0.01	1. Simple two sample t-test; 2. prevalent case.
Kurtzke et al.	1997	Caucasian	Case-control study	Veteran	23/127	9/68	13.6	14	Not significant; *P*-value not reported	1. Small sample size; 2. simple statistical analysis; 3. prevalent case.
Gustavsen et al.	2014	Caucasian	Case-control study	Population-based	391/535	391/535	13.07 (1.38)	12.97 (1.43)	0.28	1. Simple two sample *t*-test 2. prevalent case.

### Age at menarche and risk of MS–results from well-designed epidemiological investigations

During the past decade, several well-designed large-scale epidemiological investigations have been conducted, and consistent results have been reported where earlier age at menarche was found to increase the risk of MS. Ramagopalan et al. identified MS cases and controls through the population-based longitudinal Canadian Collaborative Project on Genetic Susceptibility to Multiple Sclerosis, for each index case, the spouse was taken as control, and self-reported age at puberty was used. The study collected a total of 4,472 female cases and 658 female controls (the spouse of those male cases or the same-sex partner of female cases), and assessed the effect of age of puberty through logistic regressions controlling for age at birth. The authors found that the average age at menarche for female cases was 12.4 (standard deviation = 1.29) and for female controls was 12.6 (1.33), the difference was small but statistically significant (*P* = 0.00017). Moreover, each 1-year increased age at menarche was further found to be associated with a decreased risk of MS (odds ratio = 0.90, 95% confidence interval = 0.84–0.95, *P* = 0.00063) (16).

Similarly, Nielsen et al. followed 77,330 women included in the Danish National Birth Cohort and identified 226 MS cases during an average follow-up period of 11.7 years. Information on menarcheal age was ascertained at the first interview. The authors observed a generally younger age at menarche among the cases than women without MS (13.0 vs. 13.3, *P* = 0.002). After adjusting for a number of potential confounders such as body mass index, socio-occupational status, age at first pregnancy, parity, smoking and alcohol intake, an 11% reduction in the risk of MS per 1-year increase in age at menarche was found (hazard ratio = 0.89, 95%CI = 0.81–0.98). To eliminate potential bias stemming from using self-reported data, the author further performed a supplementary analysis based on data from a subgroup of girls whom had school health records available (instead of self-reported exposure). In this subgroup a consistent reduction per 1-year increased AAM regarding MS risk was observed (OR = 0.89, 95%CI = 0.70–1.13), indicating that the potential bias due to using self-reported data was minor (17).

These results observed in European populations were further corroborated by two case-control studies conducted in Iran. Rejali et al. recruited 200 incident cases from an MS clinic and 200 sex and residential area matched controls (non-patients from the same clinic), and found a significant younger age at menarche among the cases than controls (12.96 vs. 13.48, *P* < 0.001). After controlling for the effect of age, marital status, place of residence, family history of MS, other autoimmune disease and viral disease in childhood, a significant relationship between older age at menarche and decreased risk of MS was observed (OR = 0.78, 95%CI = 0.68–0.90, *P* = 0.001) (18). Likewise, similar findings were reported by Salehi et al. examining 399 MS cases registered at the Iranian Multiple Sclerosis Society and 541 randomly selected controls from the same residential area through standard random digit dialing. The participants were interviewed and after controlling for age, marital status and education, each 1-year increase in the age at menarche was found to reduce MS risk by 10% (OR = 0.90, 95%CI = 0.82–0.98, *P* = 0.018) (19). Details of these studies are presented in Table 2.

**Table 2:** Age at menarche and risk of MS, results from well-designed epidemiological investigations.

**Author**	**Year**	**Population**	**Study**	**Design**	Cases/controls^*^	**Mean age at menarche**			**Regression model**		
						**Cases**	**Controls**	***P*-value**	**Estimate (95%CI)**	***P*-value**	**Covariates included**
Ramagopalan et al.	2008	Caucasian	case-control study	Population-based	4,472/658	12.4 (1.29)	12.6 (1.33)	0.00017	0.90 (0.84–0.95)	0.00063	Age
Nielsen et al.	2016	Caucasian	cohort study	Population-based	226/77,104	13.0 (1.5)	13.3 (1.4)	0.002	0.89 (0.81–0.98)	Not reported	BMI, socio-occupational status, age at first pregnancy, parity, smoking and alcohol intake.
Rejali et al.	2016	Caucasian (Iran)	Case-control study	Hospital-based	200/200	12.96 (1.43)	13.48 (1.49)	0.0001	0.78 (0.68–0.90)	0.001	Age, marital status, residential area, family history of MS, other autoimmune diseases and history of viral diseases in childhood.
Salehi et al.	2018	Caucasian (Iran)	Case-control study	Population-based	399/541	13.14 (1.46)	13.36 (1.67)	0.042	0.90 (0.82–0.98)	0.018	Age, marital status and education.

* *all subjects were females*.

In addition to disease status, two studies have also investigated age at menarche and age at first symptom onset specifically among MS patients. Sloka et al. examined 150 relapsing remitting female MS cases and found that age of first MS symptoms was postponed by 1.16 years as the age of menarche increased per each 1-year, using a linear regression (*r*^2^ = 0.69, *P* = 0.04). The study, however, wasn't able to account for several important confounders such as socioeconomic status or seasonal variability, and the authors argued that the induction mechanisms linking the two events together, could possibly include sharing of similar induction mechanisms, one event gives rise to another or same genetic susceptibility (20). In a recent study conducted by Bove et al. which included the major genetic risk factor of MS (HLA-DRB1^*^1501), the risk allele carriers showed an earlier age at onset (as expected), and each 1-year later age at menarche was also associated with later age at onset after adjusting for multiple potential confounders (increased by 0.63 years, P=0.033), consistent with previous findings. However, surprisingly, the MS risk allele carriers were found to have a later age at menarche than non-carriers (*P* = 0.036) (21). These results suggest the complex dynamics underlying genetics, hormonal factors and disease, as well as the importance of incorporating genetic markers when studying the complex relationship between these factors and MS.

The underlying biology on the effect of puberty on MS risk remains to be elucidated. It may regulate MS risk through multiple pathways. Firstly, experimental autoimmune encephalitis, the animal models of MS, has demonstrated a biphasic dose effect of estrogen on inflammation. At normal ranges (not pregnancy level), estrogen promotes inflammation. Thus, hormonal changes such as rise in estrogen levels after puberty may affect MS onset. In addition, puberty is also known to involve substantial brain maturational changes such as white and gray matter volume increment, and therefore plays a role in neurological modulation. Moreover, puberty appears to be a key period of exposure to some of the well-established MS risk factors, such as overweight/high body mass index, vitamin D deficiency, and Epstein-Barr virus infection. Last but not least, it is also likely that metabolic factors during puberty such as childhood nutrition, gut microbiome alterations would lead to earlier menarche and altered immunologic modulation thereby contributing to MS risk (22).

### Challenges and future directions

A powerful way to increase our knowledge on the relationship between AAM and MS, and to provide stronger evidence in support for a potentially favored role of late AAM on MS risk, is through a meta-analysis which combines results across different studies. Such analysis is currently unavailable, likely due to the limited number of well-designed and well-powered epidemiological investigations published so forth on this topic. For example, only five of the previous studies have reported both mean age at menarche and its standard deviations (15–19) and only four epidemiological studies have reported point estimates and 95% confidence intervals, three of which are case-control studies conducted in two distinct populations (16, 18, 19) and one is a cohort study (17). The heterogeneity in design and population ancestry (European or non-European) across those previous studies makes the aggregation of data difficult; and results based on data pooled from these few studies could only be considered as preliminary and suggestive. Meta-analysis or systematic review are warranted when additional well-designed large epidemiological investigations have emerged and accumulated in the future.

In addition, the observational nature of epidemiological studies could only identify association but can hardly make any conclusive causal inference, as the validity of results could be plagued by measurement error, selection bias, confounding, and reverse causality. In the case of AAM and MS, self-reported age at menarche might be inaccurate; selected clinical samples might be unrepresentative to the target population; there are several important confounders need to be taken into account, not all can be collected via conventional questionnaires; and given the long induction period of MS, certain immunological changes might already have taken place several years before the clinical diagnosis or symptom onset, information collected during this period could thus be influenced by the subclinical phase of disease itself.

Mendelian Randomization (MR) fills the gap by incorporating genetic variants (single nucleotide polymorphism, SNPs) as instrumental variables (IV) for assessing a causal effect of a risk factor on an outcome from observational data (23). A typical MR uses genetic variants (SNPs) as proxies for risk factors, rather than self-reported exposure, with the assumption that SNPs are independent of confounders in the population and randomly allocated at conception, mirroring a randomization process (as those in randomized clinical trials). Moreover, SNP allocation always precedes disease onset therefore eliminates reverse causality.

Massive investment in large-scale GWASs over the past years have discovered reliable genetic variants for a wide range of phenotypes including modifiable environmental exposures (e.g., circulating vitamin levels) and complex human behaviors (e.g., nicotine dependence), providing an unprecedented opportunity for genetic epidemiology in particular by utilizing the MR design. The success of MR approach has been demonstrated by numerous relevant works. In MS, using this approach, a causal role of vitamin D insufficiency and obesity has been strengthened (24–27). In the case of AAM, its genetic regulation has been highlighted by a recent GWAS involving 370,000 women which identified 389 independent AAM-associated signals spread over 10 biological pathways (28). A comprehensive understanding of the hypothetical causal roles for AAM in MS have therefore become possible. However, to the best of our knowledge, while MR of reproductive factors has been carried out extensively in sex-steroid-sensitive cancers with a successful identification of causal relationship (28, 29), no MR has been conducted to investigate the hypothetical causal role for pubertal development in MS to date, a sex hormone driven autoimmune disease. This is a potential future direction to be focused on, when data allows for such analysis, e.g., studies have assembled the ideal combination of large numbers of MS cases and controls, high-quality questionnaire data and high-throughput genome-wide SNPs.

In addition to investigating a putative causal relationship between AAM and MS, another direction for future research is to focus on non-European populations and males. As most of the current studies have been conducted in European ancestry populations, its generalizability has been restricted. If hormone influences MS onset, it is possible that puberty timing would also affect male MS onset through the emergence of high level sex hormones and its inhibitory effect against autoimmunity. Studying male puberty timing such as age at voice-breaking would increase our knowledge to the mechanistic developmental of MS as well as explain part of the sex disparity. Future work on other hormonal reproductive factors than AAM, and MS outcomes such as disease severity, prognosis and drug response are also warranted.

## Conclusion

In this review, we have recapitulated all the published epidemiological studies conducted so far in AAM and MS, detailing their main findings. We have illustrated the advantages and limitations of each study. There are promising evidences in support for a protective effect of late AAM on MS onset. However, the association between AAM and MS remain to be elucidated and confirmed through larger epidemiological investigations and/or meta-analysis. Future work may be conducted to focus on understanding the causal role of AAM in MS by incorporating genetic markers, from which the knowledge gained could answer some of the patients frequently interested questions in topics of hormone and disease. In addition, the broad scope of estrogen, p-pills, as well as the protection against relapses during pregnancy need further investigation. It is also important to conduct studies among non-European populations and male patient subpopulations. We anticipate that our review will inspire to activities increasing our understanding to the biological mechanisms underpinning hormonal factors and autoimmune disease MS, thus deliver meaningful implications to MS etiology.

## Author contributions

XJ reviewed the original studies conducted in the field of age at menarche and multiple sclerosis, and made the tables. XJ, TO, and LA interpreted the data, wrote and revised the manuscript.

### Conflict of interest statement

The authors declare that the research was conducted in the absence of any commercial or financial relationships that could be construed as a potential conflict of interest.
